# Astrocyte as Spatiotemporal Integrating Detector of Neuronal Activity

**DOI:** 10.3389/fphys.2019.00294

**Published:** 2019-04-18

**Authors:** Susan Yu. Gordleeva, Anastasia V. Ermolaeva, Innokentiy A. Kastalskiy, Victor B. Kazantsev

**Affiliations:** Department of Neurotechnology, Lobachevsky State University, Nizhny Novgorod, Russia

**Keywords:** astrocyte, synaptic transmission, neuron–astrocyte interaction, neuron–astrocyte network, calcium

## Abstract

The functional role of astrocyte calcium signaling in brain information processing was intensely debated in recent decades. This interest was motivated by high resolution imaging techniques showing highly developed structure of distal astrocyte processes. Another point was the evidence of bi-directional astrocytic regulation of neuronal activity. To analyze the effects of interplay of calcium signals in processes and in soma mediating correlations between local signals and the cell-level response of the astrocyte we proposed spatially extended model of the astrocyte calcium dynamics. Specifically, we investigated how spatiotemporal properties of Ca^2+^ dynamics in spatially extended astrocyte model can coordinate (e.g., synchronize) networks of neurons and synapses.

## Introduction

The functional role of astrocyte calcium signaling remains intensely debated. One of the principal reasons for such a debate is that the astrocytic Ca^2+^ dynamics possesses high complexity which was confirmed by new experimental approaches to study the signaling of astrocytes at qualitatively new spatial-temporal resolutions (Volterra et al., 2014; Bindocci et al., 2017; Wu et al., 2018). Another reason was the evidence of bi-directional astrocytic regulation of neuronal activity referred as gliotransmission [Ca^2+^-dependent release of neurotransmitters (glutamate, D-serine, ATP) by astrocytes] (Araque et al., 2014; Bazargani and Attwell, 2016; Fiacco and McCarthy, 2018; Savtchouk and Volterra, 2018).

Astrocytes respond to synaptic activity by intracellular Ca^2+^ elevations (Verkhratsky et al., 2012). Synaptically-released neurotransmitters (e.g., glutamate) can activate G-coupled receptors [e.g., the metabotropic glutamate receptors (mGluRs)] (Porter and McCarthy, 1996; Pasti et al., 1997; Perea and Araque, 2005), which, upon activation, promote inositol 1,4,5-triphosphate (IP_3_) production by phosphoinositide-specific phospholipase C β (PLCβ) (Zur Nieden and Deitmer, 2006). In turn, the elevation of cytosolic concentration of the second messenger IP_3_ promotes the Ca^2+^-induced Ca^2+^ release (CICR) from the astrocyte's endoplasmic reticulum (ER) stores. Clustering of astrocytic receptors, targeted by synaptically released neurotransmitters at points of contact of synapses with astrocytic processes (Di Castro et al., 2011; Panatier et al., 2011; Arizono et al., 2012) provides spatially confined sites of IP_3_ production, whose differential activation could result in rich spatiotemporal IP_3_ and Ca^2+^ dynamics (Volterra et al., 2014).

There are two main types of IP_3_-induced CICR observed in astrocytes (Volterra et al., 2014; Rusakov, 2015; Bindocci et al., 2017): small, fast Ca^2+^ events that are confined to their (primary) processes and caused by minimal synaptic activity; and Ca^2+^ elevations propagating along astrocytic processes that can reach the soma and trigger whole-cell global Ca^2+^ signal. The latter slow calcium events were induced by intense neuronal activity. This circumstance can be interpreted as the ability of astrocytes to perceive the information contained in the repetition rate of action potentials, however, this occurs on time scales larger than the characteristic times inherent in the synaptic activity.

The elevation of intracellular calcium concentration in astrocyte can trigger the release of various active chemicals, gliotransmitters, such as glutamate, GABA, ATP, and D-serine (Bezzi and Volterra, 2001). The concept of “tripartite synapse” (Araque et al., 1999) is based on the ability of gliotransmitters to regulate synaptic transmission and plasticity on time scales from seconds to minutes (see Araque et al., 2014 for a recent review).

Since effect of single gliotransmitter depends on the type of circuit and targeted neurons (Araque et al., 2014), we focused in our study on the excitatory transmission in hippocampus. It was shown that hippocampal astrocyte can release ATP (Zhang et al., 2003), D-serine–co-agonist of the NMDA receptor (NMDAR) (Henneberger et al., 2010; Zhuang et al., 2010) and glutamate. After conversion to adenosine, ATP can depress, or facilitate excitatory synaptic transmission acting on either A1 or A2A receptors, respectively (Serrano et al., 2006; Pascual et al., 2012). At CA3-CA1 synapses astrocytic glutamate acts on presynaptic NMDARs (Jourdain et al., 2007) or mGluRs (Navarrete and Araque, 2010; Navarrete et al., 2012) to potentiate or decrease release probability, respectively. In the CA1 region a long-term potentiation (LTP) which required presynaptic mGluR activation (Perea and Araque, 2007) can be induced by the postsynaptic activity accompanying by glutamate release from astrocyte due to Ca^2+^ elevation. Also, it was shown (Henneberger et al., 2010) that D-serine, released from astrocyte and being a coagonist of postsynaptic NMDARs, can trigger NMDAR-mediated LTP at synapses nearby of the astrocyte.

Intense modeling efforts have been devoted in recent years to understand the functional role of astrocytic modulation of the neuronal communication (see Oschmann et al., 2018 for a recent review, Kanakov et al., 2019). There are several biophysical studies of astrocytic influence on post- and presynaptic neuronal activity (De Pittà et al., 2011; Gordleeva et al., 2012; Tewari and Majumdar, 2012a,b; Tewari and Parpura, 2013; De Pittà and Brunel, 2016; Flanagan et al., 2018). However, these works did not account for the impact of spatiotemporal patterns of calcium dynamics in astrocyte, i.e., the spatial distribution of the calcium activity was neglected. Meanwhile, under the action of high-frequency action potentials in one synapse or coherent activity of several synapses, the calcium signal originating in astrocyte can spread along the processes and throughout the astrocyte and cause the release of the gliotransmitter in remote locations, affecting other synapses.

There are also a few recent computational works which investigated mechanisms underlying IP_3_-triggered CICR-mediated spatiotemporal dynamics in single astrocyte (Wu et al., 2014; Gordleeva et al., 2018; Savtchenko et al., 2018).

To analyze the principles of generation of calcium signals in processes and soma of the astrocyte and to seek mechanisms of correlations between local signals and the global signalization response of the astrocyte including its spatially distributed structure, we propose a spatially extended model of astrocyte calcium dynamics. In this paper we investigate how spatiotemporal Ca^2+^ dynamics in spatially extended astrocyte model can coordinate and synchronize network signaling.

## Methods

**Figure 1:**
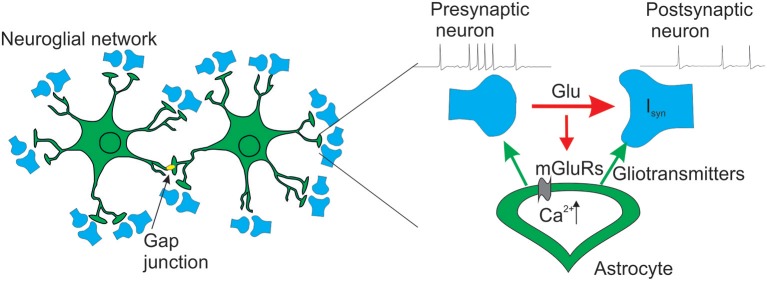
Schematic view of the neuron-astrocyte network model.

For our purpose we designed a neuron-astrocyte network composed of 100 synaptically coupled Hodgkin-Huxley excitatory neurons (Hodgkin and Huxley, 1952) and two astrocytes connected via gap junction. Each astrocyte has spatially distributed structure that repeated the morphology of real astrocyte (Bindocci et al., 2017). For illustration, we assumed that each process of the astrocyte, interacting with presynaptic and postsynaptic neurons, forms one tripartite synapse providing the connectivity between neuronal and astrocytic parts of the network. The schematic architecture of the model is shown in Figure 1. Our model of the tripartite synapse describes the effects of the astrocytic modulation of synaptic transmission in the CA1-CA3 area of hippocampus. We consider glutamate as the neurotransmitter, and glutamate and D-serine as the gliotransmitters. We describe three effects resulting from the influence of the gliotransmitters on the synapse: (i) potentiation of presynaptic release probability due to glutamate acting on presynaptic NMDARs; (ii) depression of presynaptic release probability due to glutamate acting on presynaptic mGluRs; and (iii) increase of the postsynaptic currents due to D-serine modulation of the postsynaptic NMDA receptors.

### Neural Network

Neural network consists of 100 excitatory synaptically coupled Hodgkin-Huxley neurons (Hodgkin and Huxley, 1952; Esir et al., 2018). We use random coupling topology with connection probability for each pair of neurons equal to 20% (Braitenberg and Schüz, 1998). The dynamics of the neuronal membrane potential is described by the following ionic current balance equation:

(1) $$\begin{array}{l} C\frac{dV^{\left(n\right)}}{dt}=I_{channel}^{\left(n\right)}+I_{app}^{\left(n\right)}+\underset{m}{\sum }I_{syn}^{\left(mn\right)}+I_{P}^{\left(n\right)}, \end{array}$$

where capacitance, *C*, is 1 μF/cm^2^, the superscript *(n* = *1,…, M)* corresponds to a neuronal index and *(m)* corresponds to an index of input connection. The Na^+^, K^+^, and leak currents are expressed as follows:

(2) $$\begin{array}{l} I_{channel}=-g_{Na}m^{3}h\left(V-E_{Na}\right){-g}_{K}n^{\;4}\left(V-E_{K}\right)\\ \;-g_{leak}\left(V-E_{leak}\right), \end{array}$$

where *g*_*Na*_ and *g*_*K*_ are the potassium and sodium conductances (mS/cm^2^), *E*_*Na*_, and *E*_*K*_ are the potassium and sodium reversal potentials (mV), *g*_*leak*_ and *E*_*leak*_ are the leak conductances and leak reversal potential, respectively.

The kinetics of the potassium channel is determined by:

(3) $$\begin{array}{l} \frac{dm}{dt}=\alpha_{m}\left(1-m\right)-\beta_{m}m,\\ \alpha_{m}=\frac{0.1\left(V+40\right)}{1-\exp\left(-\left(V+40\right)/10\right)},\\ \beta_{m}=4\exp\left(-\left(V+65\right)/18\right), \end{array}$$

(4) $$\begin{array}{l} \frac{dh}{dt}=\alpha_{h}\left(1-h\right)-\beta_{h}h,\\ \alpha_{h}=0.07\exp\left(-\left(V+65\right)/20\right),\\ \beta_{h}=\frac{1}{1+\exp\left(-\left(V+35\right)/10\right)}. \end{array}$$

The kinetics of the sodium channel is determined by:

(5) $$\begin{array}{l} \frac{dn}{dt}=\alpha_{n}\left(1-n\right)-\beta_{n}n,\\ \alpha_{n}=\frac{0.01\left(V+55\right)}{1-\exp\left(-\left(V+\;55\right)/10\right)},\\ \beta_{n}=0.125\exp\left(-\left(V+65\right)/80\right). \end{array}$$

The applied currents *I*${\;}_{app}^{\left(n\right)}$ are fixed at constant value controlling the depolarization level and dynamical mode of the neuron (Kazantsev and Asatryan, 2011). We use *I*${\;}_{app}^{\left(n\right)}$ = 4.5 μA/cm^2^ which corresponds to the neuron's excitable mode. The synaptic current *I*_*syn*_ (μA/cm^2^) simulating interactions between the neurons. Each neuron is stimulated by a Poisson pulse train mimicking external spiking inputs *I*${\;}_{P}^{\left(n\right)}$ (μA/cm^2^) with a certain average rate λ. Each Poisson pulse has rectangular shape with fixed width of 10 ms and variable height, which is sampled independently for each pulse from uniform random distribution on interval [0, 1.5]. Sequences of Poisson pulses applied to different neurons are independent.

### Synaptic Dynamics

Each spike on presynaptic neuron results in the release of the glutamate quant. We describe presynaptic dynamics of the glutamate, *G*, using a mean field approach from Gordleeva et al. (2012):

(6) $$\begin{array}{l} \frac{dG}{dt}=-\alpha_{G}\left(G-k_{pre}\delta \left(t-t_{k}\right)\right), \end{array}$$

where α_*G*_ denotes the clearance rate of the neurotransmitter, *k*_*pre*_ denotes the efficacy of the presynaptic release, δ denotes the Dirac delta function and *t*_*k*_ is spike time.

The release of the glutamate leads to excitatory postsynaptic current (EPSC). For description of the EPSCs dynamics we use the approach from our previous work (Gordleeva et al., 2012):

(7) $$\begin{array}{l} \frac{dI_{EPSC}}{dt}=-\alpha_{I}\left(I_{EPSC}-A\delta \left(t-t_{k}\right)\right),\\ P\left(A\right)=\frac{2A}{b^{2}}\exp\left(-\frac{A^{2}}{b^{2}}\right),\int_{0}^{+\infty }P\left(A\right)dA=\Gamma \left(1\right)=1, \end{array}$$

where α_*I*_ is rate constant. According to the experimental data (Fernández-Ruiz et al., 2012; Guzman et al., 2016) we supposed that amplitude of the EPSCs, *A*, is gamma-distributed with probability density function *P(A)*, where *b* is the scaling parameter of gamma-distribution that denotes the impact of the synaptic input.

Integrated synaptic current of the neuron, *I*_*syn*_, is described by the following equation (Gordleeva et al., 2012):

(8) $$\begin{array}{l} I_{syn}=\frac{I_{EPSC}}{1+\exp\left(-\left(G-\theta_{G}\right)/k_{G}\right)}, \end{array}$$

where θ_*G*_ denotes the midpoint and *k*_*G*_ denotes the slope of the neuronal activation function.

### Geometry of the Astrocytic Model and Astrocytic Ca^2+^ and IP_3_ Dynamics

**Figure 2:**
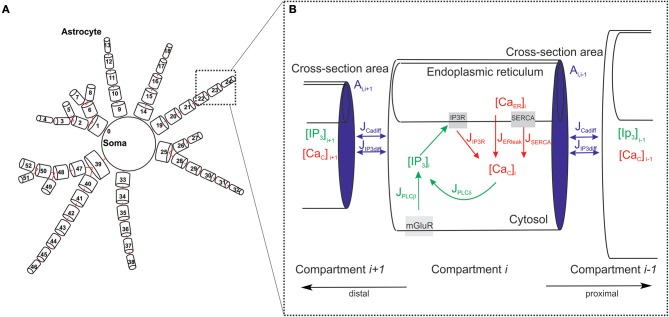
Geometry of the astrocytic model. **(A)** Schematic view of the spatially distributed astrocyte model. **(B)** Model of the astrocyte dynamics. Schematic representation of basic IP_3_ and Ca^2+^ currents and their kinetics taken into account for each compartment.

To design the architecture of the spatially distributed astrocyte model, we followed available experimental facts (Bindocci et al., 2017) (see Figure 2A). Specifically, we consider the astrocyte as network of inter-coupled small compartments, which have a cylindrical shape (Gordleeva et al., 2018). Each element is a unit-length cylinder with a finite radius *r* containing ER (Figure 2B). Compartments are coupled through the diffusion of calcium and IP_3_ controlling the calcium exchange between the cytoplasm and intracellular stores of calcium (in particular, ER).

The dynamics of each compartment is described by the following set equations (modified from Li and Rinzel, 1994; Gordleeva et al., 2018). The balance of calcium fluxes in cytosol for particular compartment is described by

(9) $$\begin{array}{l} \frac{d{\left[Ca_{c}\right]}_{i}}{dt}=\frac{S_{ERi}}{F\cdot V_{i}}\left(J_{IP3R}-J_{SERCA}+J_{ERleak}\right)+J_{Cadiff}. \end{array}$$

Changing free calcium concentration in the cytosol of the compartment *i* is defined by calcium exchange with the ER involving the calcium release from the ER to the cytosolic volume through IP_3_ receptors, *J*_*IP*3*R*_, the ATPase Ca^2+^- pump “SERCA” by *J*_*SERCA*_, and by calcium leak from the ER, *J*_*ERleak*_. *S*_*ERi*_ = *S*_*i*_$\sqrt{r_{ERi}}$ denotes the surface of the ER. The volume and the surface of the intracellular space are defined as *V*_*i*_ and *S*_*i*_, respectively. *r*_*ER*_ is the ratio of the volume of ER to the volume of cytoplasm in the considered compartment of the astrocyte. The distribution of values over compartments in the developed model was chosen according to experimental data (Patrushev et al., 2013; Oschmann et al., 2017) and can be found in Supplementary Table 2.

The current *J*_*IP*3*R*_ is expressed by the following equations (Li and Rinzel, 1994) approximating the kinetics of ER IP_3_Rs:

(10) $$\begin{array}{l} J_{IP3R}=\frac{FV_{i}}{S_{i}}v_{1}m_{\infty }^{3}n_{\infty }^{3}h^{3}\left({\left[Ca_{ER}\right]}_{i}-{\left[Ca_{c}\right]}_{i}\right),\\ \frac{dh_{i}}{dt}=\frac{h_{\infty }-h_{i}}{\tau_{h}},\\ h_{\infty }=d_{2}\frac{\left({\left[IP_{3}\right]}_{i}+d_{1}\right)\left({\left[IP_{3}\right]}_{i}+d_{3}\right)}{\left(d_{2}\left({\left[IP_{3}\right]}_{i}+d_{1}\right)+{\left[Ca_{c}\right]}_{i}\left({\left[IP_{3}\right]}_{i}+d_{3}\right)\right)},\\ n_{\infty }=\frac{{\left[Ca_{c}\right]}_{i}}{{\left[Ca_{c}\right]}_{i}+d_{5}},m_{\infty }=\frac{{\left[IP_{3}\right]}_{i}}{{\left[IP_{3}\right]}_{i}+d_{1}},\\ \tau_{h}=\frac{\left({\left[IP_{3}\right]}_{i}+d_{3}\right)}{a_{2}\left(d_{2}\left({\left[IP_{3}\right]}_{i}+d_{1}\right)+{\left[Ca_{c}\right]}_{i}\left({\left[IP_{3}\right]}_{i}+d_{3}\right)\right)}. \end{array}$$

Here *[Ca*_*c*_*]*_*i*_ and *[Ca*_*ER*_*]*_*i*_ are calcium concentrations in the cytosol and in the ER of the compartment *i*, respectively. The dynamics of the Ca^2+^ concentration in the ER is described by:

(11) $$\begin{array}{l} \frac{d{\left[Ca_{ER}\right]}_{i}}{dt}=\frac{S_{ERi}}{F\cdot V_{ERi}}\left(-J_{IP3R}+J_{SERCA}-J_{ERleak}\right)+J_{CaERdiff} \end{array}$$

where *S*_*ERi*_ and *V*_*ERi*_ denote the area and the volume of the ER, respectively.

The variable *h* denotes the fraction of activated IP_3_ receptors and the other gating variables for IP_3_Rs are set to their equilibrium values *m*_∞_ and *n*_∞_. Active ATP-dependent current *J*_*SERCA*_ pumping calcium back to the ER and the Ca^2+^ leak current from the ER, *J*_*ERleak*_, are given by the following equations:

(12) $$\begin{array}{l} J_{SERCA}=\frac{FV_{i}}{S_{i}}v_{3}\frac{{{\left[Ca_{c}\right]}_{i}}^{2}}{{{\left[Ca_{c}\right]}_{i}}^{2}+k_{3}^{2}},\\ J_{ERleak}=\frac{FV_{i}}{S_{i}}v_{2}\left({\left[Ca_{ER}\right]}_{i}-{\left[Ca_{c}\right]}_{i}\right). \end{array}$$

The change of the IP_3_ concentration is defined by production and degradation that are regulated by cytosolic Ca^2+^. These include Ca^2+^-dependent phospholipase C δ (PLCδ) mediated IP_3_ synthesis and Ca^2+^-dependent IP_3_ degradation by the IP_3_ 3-kinase (IP_3_-3K) and the inositol polyphosphate 5-phosphatase (IP-5P) (De Pitta et al., 2009):

(13) $$\begin{array}{l} \frac{d{\left[IP_{3}\right]}_{i}}{dt}=J_{PLC\beta }+J_{PLC\delta }-J_{\deg3K}-J_{\deg5P}+J_{IP3diff}. \end{array}$$

The first current, *J*_*PLCβ*_, describes agonist dependent IP_3_ production by PLCβ. The activation of PLCβ by G-protein is controlled by the glutamate concentration, *G*, (Equation 6) (De Pitta et al., 2009):

(14) $$\begin{array}{l} J_{PLC\beta }=v_{\beta }\frac{G^{0.7}}{G^{0.7}+{\left(K_{R}+K_{p}\cdot \frac{{\left[Ca_{c}\right]}_{i}}{{\left[Ca_{c}\right]}_{i}+K_{\pi }}\right)}^{0.7}}, \end{array}$$

where *v*_β_ is the rate of IP_3_ production by PLCβ*, K*_*R*_ is the glutamate affinity of the receptor, *K*_*p*_ is Ca^2+^/PLC- dependent inhibition constant, and *K*_π_ is Ca^2+^ affinity of PLC.

The second term in Equation (13) describes cytosolic calcium dependent PLCδ activation (De Pitta et al., 2009):

(15) $$J_{PLC\delta }=\frac{v_{\delta }}{1+\frac{{\left[IP_{3}\right]}_{i}}{k_{\delta }}}\frac{{\left[Ca_{c}\right]}_{i}{\;}^{2}}{{\left[Ca_{c}\right]}_{i}{\;}^{2}+K_{PLC\delta }^{2}},$$

where *v*_δ_ described the maximal rate of IP_3_ production by PLCδ*, k*_δ_– the inhibition constant, and *K*_*PLCδ*_– the Ca^2+^ affinity of PLCδ.

The IP_3_ degradation by IP_3_-3K and IP-5P described by the following equations (De Pitta et al., 2009):

(16) $$\begin{array}{l} J_{\deg3K}=v_{3K}\frac{{{\left[Ca_{c}\right]}_{i}}^{4}}{{{\left[Ca_{c}\right]}_{i}}^{4}+K_{D}^{4}}\cdot \frac{{\left[IP_{3}\right]}_{i}}{{\left[IP_{3}\right]}_{i}+K_{3}},J_{\deg5P}=r_{5P}{\left[IP_{3}\right]}_{i}. \end{array}$$

The whole process dynamics is formed by the intracellular diffusion of calcium and IP_3_ accounted by the following fluxes:

(17) $$\begin{array}{l} J_{IP3diff}=d_{IP3\left(i,i+1\right)}\left({\left[IP_{3}\right]}_{\left(i+1\right)}-{\left[IP_{3}\right]}_{i}\right)\\ \;+d_{IP3\left(i,i-1\right)}\left({\left[IP_{3}\right]}_{\left(i-1\right)}-{\left[IP_{3}\right]}_{i}\right),\\ J_{Cadiff}=d_{Ca\left(i,i+1\right)}\left({\left[Ca_{c}\right]}_{\left(i+1\right)}-{\left[Ca_{c}\right]}_{i}\right)\\ \;+d_{Ca\left(i,i-1\right)}\left({\left[Ca_{c}\right]}_{\left(i-1\right)}-{\left[Ca_{c}\right]}_{i}\right). \end{array}$$

The diffusion flux through compartments of ER described by the following:

(18) $$\begin{array}{l} J_{CaERdiff}=d_{CaER}\left({\left[Ca_{ER}\right]}_{\left(i+1\right)}+{\left[Ca_{ER}\right]}_{\left(i-1\right)}-2{\left[Ca_{ER}\right]}_{i}\right). \end{array}$$

Note, that the values of the diffusion rates from compartment *j (j* = *i* + *1; j* = *i* − *1)* to compartment *i* for IP_3_, *d*_*IP*3*ij*_, and for calcium, *d*_*Caij*_, depend on the compartment geometry (e.g., compartment volume) and are different the inward and outward fluxes at the process branching sites:

(19) $$\begin{array}{l} d_{IP3ij}=\frac{D_{IP3}A_{ij}}{V_{i}\cdot x_{ij}},d_{Caij}=\frac{D_{Ca}A_{ij}}{V_{i}\cdot x_{ij}}, \end{array}$$

where *A*_*ij*_ is the cross-section area between compartments, *V*_*i*_ is the volume of compartment *i, x*_*ij*_ is the distance between the centers of the nearest-neighbor compartments, which is equal to the unite length of compartment. *D*_*IP*3_ and *D*_*Ca*_ is the diffusion constant for IP_3_ and Ca^2+^, respectively. For simplicity, we assume that the size of the ER is the same in all compartments and therefore the diffusion coefficient of Ca^2+^ in the ER does not depend on the geometry of the compartment and is constant. Values of model parameters can be found in Supplementary Table 1. Note that the time unit in the neuronal model (1–5) is 1 ms. Due to a slower time scale, in the astrocytic model empirical constants are indicated using seconds as time units. When integrating the joint system of differential equations, the astrocytic model time is rescaled so that the units in both models match up.

### Dynamics of Gliotransmitters

When the *[Ca*_*c*_*]* in astrocytic processes exceeds a threshold *[Ca*_*c*_*]*_*th*_ concentration, gliotransmitter is released by the astrocyte into the extra-synaptic space. For illustration, we consider that the gliotransmitter is released only from the distal compartment on each astrocytic process forming the tripartite synapse. We describe the concentration of gliotransmitter by the following equations (Gordleeva et al., 2012):

(20) $$\begin{array}{l} \frac{dY_{k}}{dt}=-\alpha_{k}\left(Y_{k}-H_{k}\left(\left[Ca_{c}\right]\right)\right),\\ H_{k}\left(\left[Ca_{c}\right]\right)=\frac{1}{1+\exp\left(-\frac{\left[Ca_{c}\right]-{\left[Ca_{c}\right]}_{th}}{k_{k}}\right)}, \end{array}$$

where index *k* denotes the type of gliotransmitter released from astrocyte: *k* = 1 for glutamate and *k* = 2 for D-serine. α_*k*_ denotes the gliotransmitter clearance rate. The amount of the gliotransmitter released from astrocyte if the Ca^2+^ concentration exceeds a threshold accounted by the function *H*_*k*_*([Ca*_*c*_*])*.

Glutamate released from astrocyte can modulate presynaptic release. Equation for presynaptic dynamics (6) considering the astrocytic modulation should be modified to:

(21) $$\begin{array}{l} \frac{dG}{dt}=-\alpha_{G}\left(G-k_{0}\left(1+\gamma_{1}Y_{1}\right)\delta \left(t-t_{k}\right)\right), \end{array}$$

where the influence of glutamate released from astrocyte on the amount of neurotransmitter describes by parameter γ_1_. γ_1_ > 0 for the potentiation and γ_1_ < 0 for the depression of neurotransmitter release, respectively.

Astrocytic D-serine modulates the response of the NMDARs on the postsynaptic terminal. This modulation leads to increase the amplitudes of postsynaptic currents. In the model it means the increase of the scaling parameter, *b*, of the probability density function *P(A)* (7):

(22) $$\begin{array}{l} b=b_{0}\left(1+\gamma_{2}Y_{2}\right), \end{array}$$

where γ_2_ is the parameter which describe impact of the astrocytic D-serine on the amplitudes of the EPSCs.

## Results

**Figure 3:**
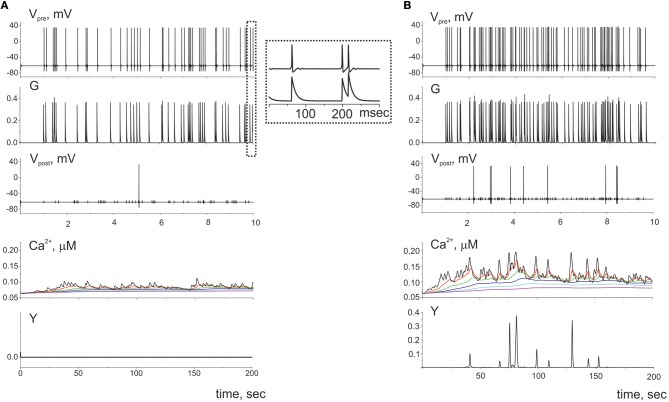
The dynamics of the tripartite synapse without astrocytic influence on synaptic transmission for two frequencies of spiking presynaptic neuron: **(A)** 5 Hz and **(B)** 10 Hz. The *G(t)* is the mean field concentration of glutamate released for each spike on the presynaptic neuron (*V*_*pre*_*(t)*). Released into synaptic cleft glutamate induced firing of the postsynaptic neuron (*V*_*post*_*(t)*) and rise of Ca^2+^ in the cytosol of the perisynaptic process compartment (*Ca*^2+^*(t)*). The elevation of intracellular concentration of Ca^2+^ in the astrocytic compartment trigger release gliotransmitter (*Y(t)*) and can propagate along the process to the soma. Time realizations of the intracellular calcium concentrations are marked by the different color in different compartments of the process and shown the propagation of the calcium signals. Axis designation *(Ca*^2+^*)* corresponds to the model variable *[Ca*_*c*_*]* described by Equation (9).

First, let us consider the dynamics of single tripartite synapse without influence of the gliotransmitters on the synaptic strength. The dynamics of synaptic transmission obtained in model (1–20) is shown in Figure 3. We consider quite low frequency of presynaptic firing (Figure 3A). The model has been tuned to follow recent experimental data on the calcium activity of astrocyte taken *in vivo* on a subcellular scale (Bindocci et al., 2017). They showed that astrocyte could response even on the low frequency of neuronal activity. According to experimental data the parameters values were chosen so that even individual action potential on the presynaptic neuron could induce small calcium event in the most distant astrocytic compartment. In response to the glutamate release from presynaptic terminal, the calcium concentration in the distal compartment increases. This increase, however, is not sufficient to trigger the gliotransmission (e.g., *Y(t)* is close to zero), because the intracellular diffusion calcium concentration consequently increases in all elements of this process. However, the amplitude of these pulses is smaller than the amplitude of the basic response. With increase of presynaptic firing rate the amplitudes of the calcium signals in the distal compartment substantially increase and exceed the threshold of gliotransmission (Figure 3B).

**Figure 4:**
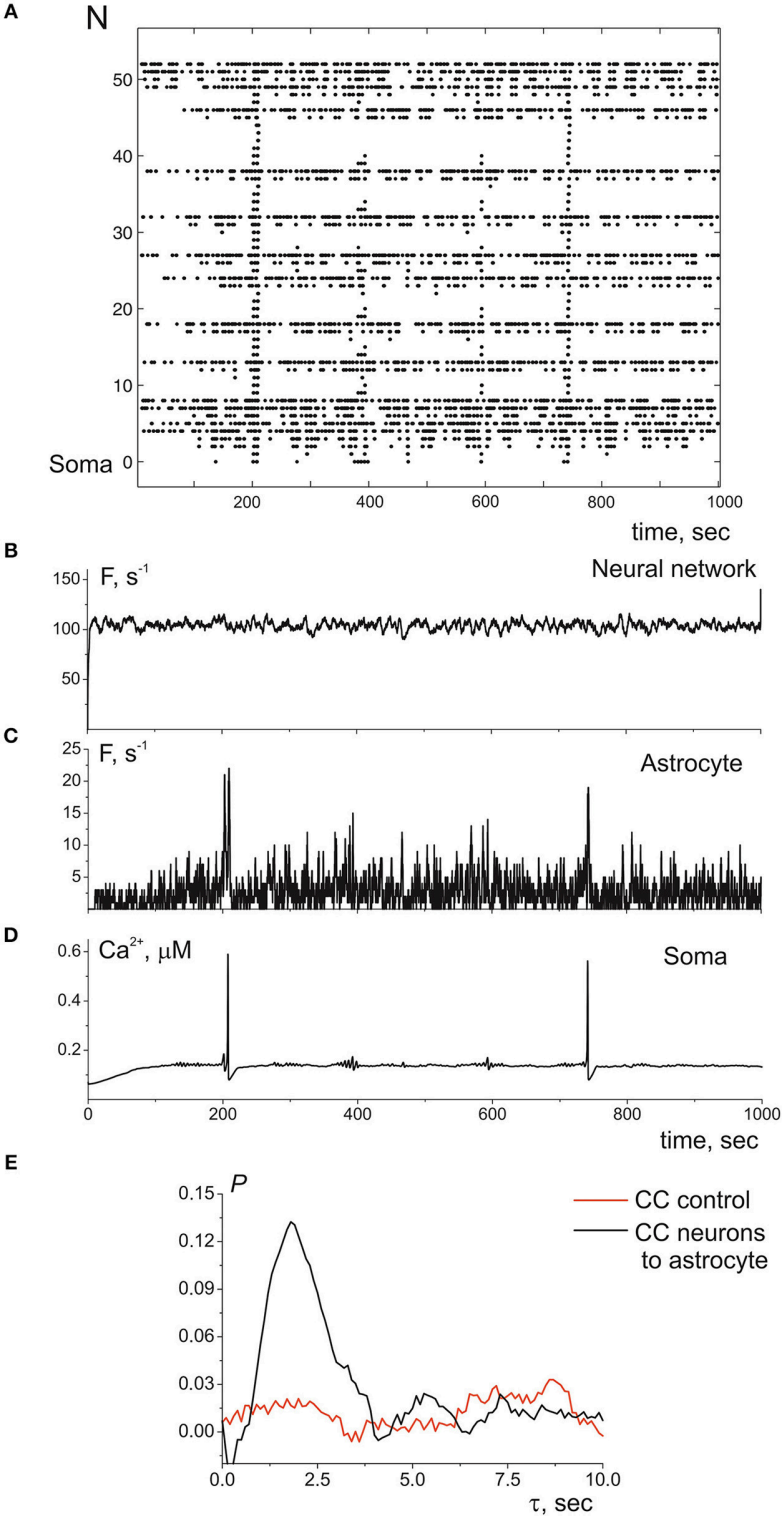
**(A)** A raster plot of the calcium activity in astrocyte, where each dot represents a calcium signal (increase of Ca^2+^ concentration in compartment above threshold in 0.15 μM). **(B)** The neuronal firing rate, i.e., the number of spikes in the presynaptic neurons in the 100-ms time window. **(C)** The calcium firing rate in astrocyte, the number of Ca^2+^ signals in the 100-ms time window. **(D)** The time realization of Ca^2+^ concentration in soma. **(E)** The cross correlation between **(B,C)**—black line. The cross correlation between **(C)** and firing rate of all neuronal network—red line. λ = 9.3 Hz.

**Figure 5:**
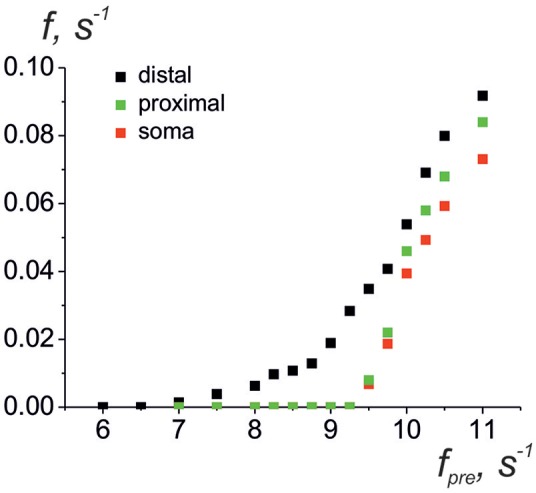
Average frequencies of generation of calcium signals in soma (red dots), in all distal processes (black dots), and in all proximal processes the nearest to soma (green dots) vs. the frequency of the presynaptic neurons.

Next, we consider the interaction of whole astrocyte and neural network. All neurons of the network are stimulated by Poisson process with fixed frequency. The model architecture of the astrocyte model includes 14 processes, and, hence, the astrocyte interacts with 14 synapses from neural network of 36 neurons. Figure 4A illustrates the space-time diagram of calcium signals in the compartmental model. Note that the frequency of calcium signals in soma is much lower than in all compartments of the astrocyte. Calcium signals generated in the distal elements of different processes of the astrocyte propagate to the soma due to diffusion. An increase in the calcium concentration in the soma of the cell induces the propagation of the Ca^2+^ signal back through all processes of the model. If one compares the astrocytic calcium activity firing rate (the number of calcium signals in all compartments in the 100-ms time window) (Figure 4C) and corresponding time trace of the intracellular calcium concentration in soma (Figure 4D), then it happens that the calcium response in the soma occurs as the result of a space–time integration of calcium fluctuations in the processes of the astrocyte. To estimate the level of functional connectivity between activity of neurons and calcium activity in astrocyte, we calculated the cross correlation (CC) between neuronal firing rate (Figure 4B) and astrocytic firing rate (Figure 4C). The peak of the CC (Figure 4E) indicates the presence of cross correlation (e.g., a synchrony) and estimates the communication delay time τ equal about to 2 s. It is important to note that the peak of the CC exists only for firing rate of presynaptic neurons not all neurons in network (red line on the Figure 4E). Thus, the model verified that activation of the astrocyte is stimulated by synchronous in time and in space neuronal activity. This correspond to the experimental data presented in Bindocci et al. (2017). They found very large events *in vivo*, which they called global Ca^2+^ events, that spread spatially to most of the astrocytic structures. Most of global calcium events were registered during movement of the mouse associated intense neuronal discharges. The dependences of the average frequencies of generation of calcium signals in the soma and processes of the astrocyte on the firing frequency of the presynaptic neurons are shown on the Figure 5. Note that the frequency at the distal compartments reaches the highest values being monotonically dependent on the firing rate of presynaptic neurons and accordingly on the release rate of the neurotransmitter. Calcium signals on the astrocyte soma occur less often when exceeding a certain threshold of the spiking frequency of the presynaptic neurons.

**Figure 6:**
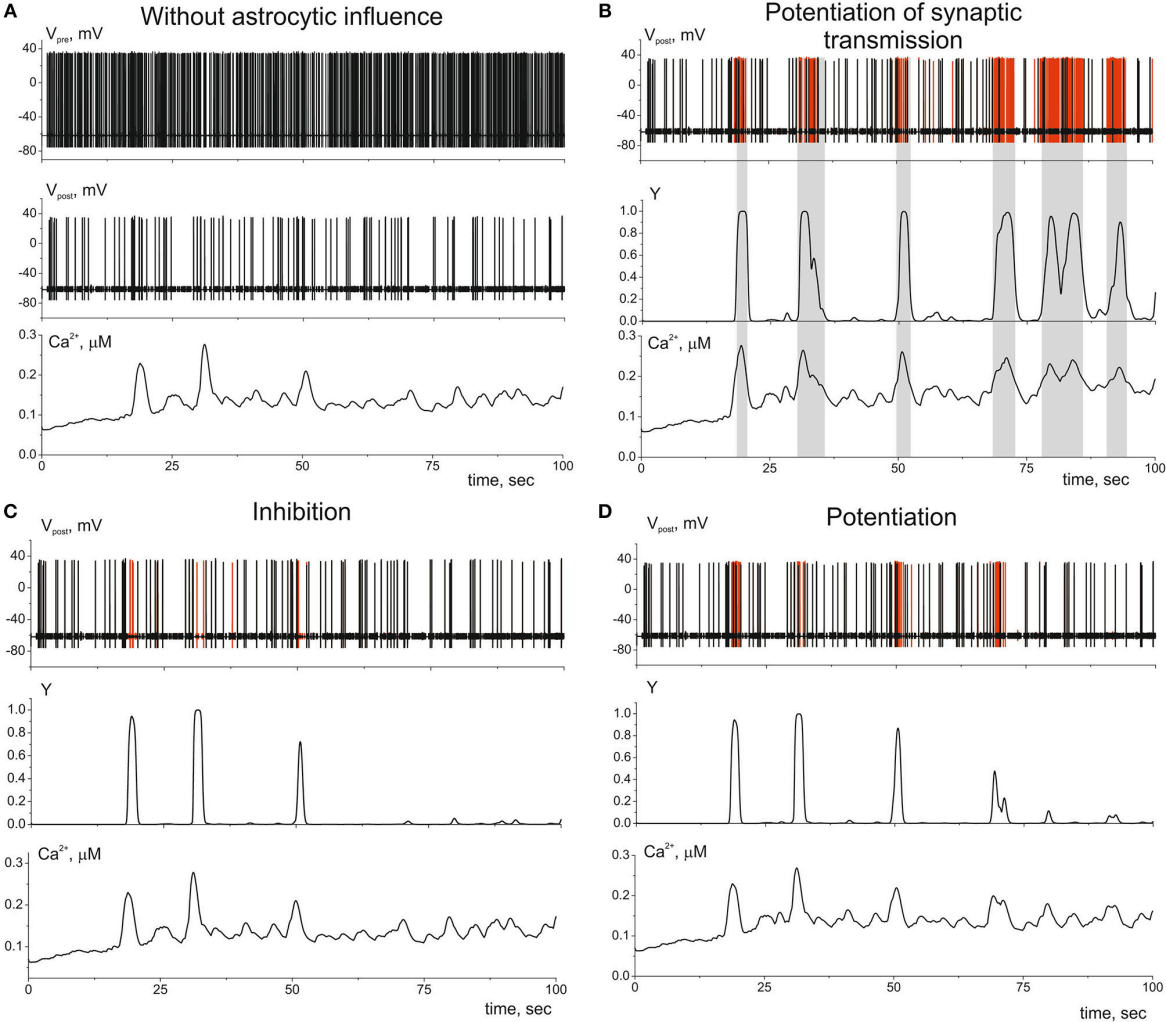
The dynamics of the tripartite synapse with astrocytic influence on synaptic transmission. **(A)** Time traces of membrane potentials of pre- (*V*_*pre*_*(t)*) and post-synaptic (*V*_*post*_*(t)*) neurons and calcium concentration (*Ca*^2+^*(t)*) in distal compartment of the astrocytic process without impact of astrocyte (γ_1_ = γ_2_ = 0). **(B)** Astrocyte-mediated potentiation of presynaptic release. Red line corresponds to the post-synaptic activity with astrocytic influence (γ_1_ = 0.1). Black—without. *Y(t)* is the time trace of gliotransmitter concentration. **(C)** Astrocyte-mediated inhibition of presynaptic release. Red line corresponds to the postsynaptic activity without astrocytic influence (γ_1_ = −0.4). **(D)** Astrocyte-mediated increasing of PSCs amplitudes. Red line corresponds to the postsynaptic activity with astrocytic influence (γ_2_ = *1*). λ = 9 Hz.

Let us now consider the impact of gliotransmitter on the synaptic transmission. Figure 6 shows dynamics of single tripartiate synapse. We stimulate the presynaptic neuron by Poisson process and register the activities of postsynaptic neuron, gliotransmitter, and Ca^2+^ concentration in perisynaptic process. We analyze the following astrocyte-mediated modulations of synaptic transmission: (i) astrocytic glutamate potentiates of neurotransmitter release by acting on presynaptic NMDARs, γ_1_ > 0 in (21) (Jourdain et al., 2007) (Figure 6B), (ii) astrocytic glutamate can target presynaptic mGluRs which decrease release probability, γ_1_ < 0 in (21) (Semyanov and Kullmann, 2000; Perea and Araque, 2007; Navarrete et al., 2012) (Figure 6C); (iii) the gliotransmitter D-serine triggers increase of the amplitudes of post synaptic currents by acting as the coagonist of postsynaptic NMDARs, γ_2_ > 0 in (22) (Henneberger et al., 2010) (Figure 6D).

**Figure 7:**
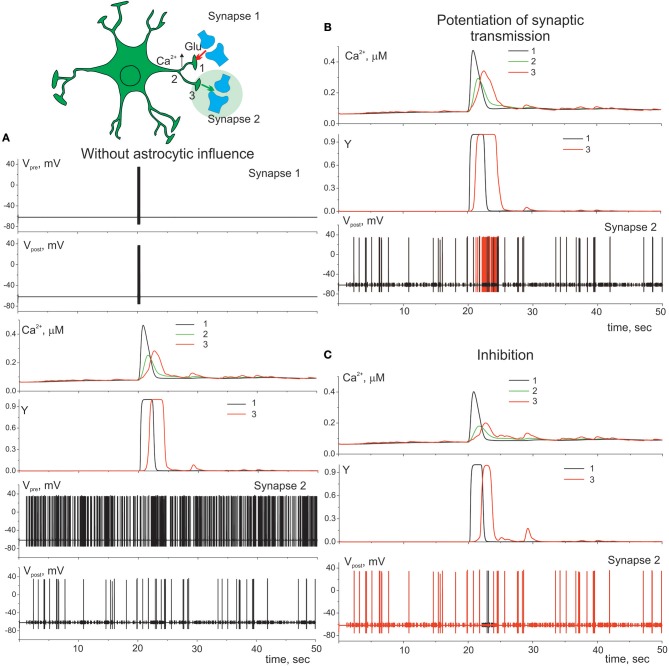
Heterosynaptic astrocyte-induced modulation of transmission. **(A)** Presynaptic neuron (synapse 1) is stimulated by the short high-frequency discharge. That triggers elevation of calcium in compartment 1 of astrocyte which spread to the other compartments 2, 3 of the process. Presynaptic neuron (synapse 2) is stimulated by the Poisson process. λ = 9 Hz. Potentiation **(B)** (γ_1_ = 0.1) and inhibition **(C)** (γ_1_ = –0.4) of presynaptic release in synapse 2. Red color corresponds to the membrane potential of the postsynaptic neuron (synapse 2) with astrocyte impact, black color—without.

Gliotransmission can co-ordinate several synapses and networks of neurons (Serrano et al., 2006; Pascual et al., 2012). Figure 7 illustrates the heterosynaptic astrocyte-induced modulation of neurotransmission in our model. Ca^2+^ signals evoked locally by high-frequency discharge of synapse 1 (Figure 7A) can propagate intracellularly from their initial source toward different process and trigger gliotransmitter release at nearby synapse (synapse 2) facilitating (Figure 7B) or depressing (Figure 7C) synaptic transmission.

**Figure 8:**
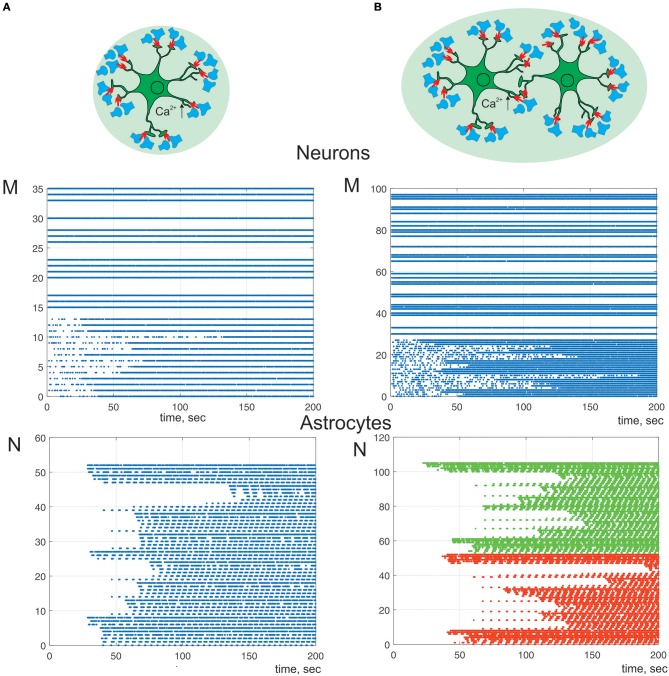
A raster plots of the neuronal and calcium activities in astrocyte. λ = 7 Hz. We consider astrocyte-mediated potentiation of presynaptic release (γ_1_ = 0.1). Neuron-astrocyte network under study consists of **(A)** one astrocyte and 36 neurons. Neurons 0–14 are postsynaptic; **(B)** two connected via gap-junction astrocytes (color of dots correspond to different cells) and 100 neurons. Neurons 0–28 are postsynaptic.

Next, we study a bidirectional regulation of the signal transmission in neural ensemble by astrocytes. According to the experimental data astrocytes occupy non-overlapping territories (Halassa et al., 2007). Thus, only one astrocyte can impact on transmission in a determined set of thousands synapses. We consider neuron-astrocyte network consists of two astrocytes connected via gap junction and 100 synaptically coupled neurons (Figure 8). Figure 8A shows communication of one astrocyte and small neuronal network of 36 neurons the same as on Figure 4 but with taking into consideration astrocytic modulation of synaptic transmission. High frequency of neuronal firing rate increases the probability of synchronous activity in neighboring to astrocyte synapses. Such coherent synaptic activity induces Ca^2+^ elevations in different astrocytic processes, which due to spatial-temporal integration results in global, long lasting Ca^2+^ events. This large calcium signal reaching the cell soma and triggering whole-cell Ca^2+^ signaling can stimulate the release of gliotransmitter to multiple synapses coordinating activity of the neuronal circuit (Figure 8A) defined by the morphological territory of individual astrocyte. Thus, astrocyte-mediated potentiation of presynaptic release results in long-term increasing of neuronal activity from domain defined by the astrocytic morphology (Figure 8A). This kind of response can even propagate to neighboring astrocytes, through gap junction channels. Figure 8B shows the simulation of the calcium signal propagation through the processes of one astrocyte to another triggering modulation of communication in large neuronal ensembles.

## Discussion

The majority of known data is extracted from Ca^2+^ signals monitoring in astrocyte soma. Slow Ca^2+^ events in astrocyte used to be associated with the high level of neuronal activity (Pasti et al., 1997; Sul et al., 2004). Recent studies, indeed, revealed that even a minimal synaptic activity is capable of small, rapid, and localized Ca^2+^ response excitation in astrocyte (Volterra et al., 2014; Bindocci et al., 2017). These data gave a ground to assume that astrocytes generate large calcium signal by integrating the activity of several individual synapses. Thus, the astrocyte Ca^2+^ signaling represents self-coordinated spatio-temporal patterns including local fast responses as well as, respectively, slow global responses resulting from the integration of the signals. The integration can encapsulate the mechanism of the global responses control via local changes in neuronal activity.

Our model accounting for spatial morphology of the tripartite synapses revealed interesting functional features of calcium activity in astrocyte-mediated modulation of signal transmission. It was shown that astrocyte can act as temporal and spatial integrator, hence, detecting the level of spatio-temporal coherence in the activity of accompanying neuronal network. Specifically, such time and space integration based on rapid and local events of activation of small compartments along the astrocytic processes results in the long-term astrocyte-mediated changes of the synaptic functionality of the neuronal network. Revealed by a correlation analysis of obtained numerical simulations, a presence of the synchrony between neuronal and astrocytic activity has verified that activation of the astrocyte is stimulated by neuronal activity, which is synchronous in time and in space.

In this study we show that different level of the neuronal activity can trigger Ca^2+^ dynamics in astrocyte with various spatio-temporal characteristics which can lead to different astrocytic-induced regulatory effects on synaptic transmission. The minimal synaptic activity causes the fast and local Ca^2+^ elevation in astrocytic process. This small Ca^2+^ signal triggers the gliotransmission in the active synapse induces localized regulatory astrocytic feedback of the synapse (Figure 6). Increasing frequency of synaptic activity can produce Ca^2+^ signal which can spread to another astrocytic process (Figure 7) and to the whole cell (Figure 8). Therefore, it can result in modulation of activity in neighboring synapses (Figure 7) and domain of synapses restricted by the territory of astrocytic morphology (Figure 8). In other words astrocyte can induce spatial synchronization in neuronal circuits defined by the morphological territory of the astrocyte. It is known that spatial synchronization in the brain is responsible for various cognitive functions (attention, recognition, navigation, making decisions, etc.) and for various pathologies (epileptic discharges, etc.).

## Author Contributions

SG, AE, and VK: conceptualization. SG and AE: data curation. SG, AE, and IK: formal analysis, investigation, and software. SG and VK: supervision and writing original draft.

### Conflict of Interest Statement

The authors declare that the research was conducted in the absence of any commercial or financial relationships that could be construed as a potential conflict of interest.
