# Supplementary material for: Astrocyte as Spatiotemporal Integrating Detector of Neuronal Activity
Source: Front Physiol. 2019 Apr 18;10:294. doi: 10.3389/fphys.2019.00294 (PMC6482266; doi:10.3389/fphys.2019.00294)
Supplement: Supplementary file 1 [file Table_1.pdf]

**Table 1. The parameter values used in the model**

| Parameter | Definition | Value | References |
|---|---|---|---|
| $g_{Na}$ | Conductivity of Na current | 120 mS/sm$^2$ | [1] |
| $g_K$ | Conductivity of K current | 36 mS/sm$^2$ | [1] |
| $g_{leak}$ | Leakage current conductivity | 0.3 mS/sm$^2$ | [1] |
| $V_{Na}$ | Reversible Na potential | 55 mV | [1] |
| $V_K$ | Reversible K potential | -77 mV | [1] |
| $V_{leak}$ | Reversible leakage potential | -54.4 mV | [1] |
| $C$ | Specific membrane capacity | 1 μF/sm$^2$ | [1] |
| $I_{app}$ | Current simulating constant membrane depolarization | 4.5 μA/sm$^2$ | [1, 2] |
| $\alpha_G$ | Neurotransmitter clearance constant | 0.1 ms$^{-1}$ | [3] |
| $\alpha_I$ | Rate constant of EPSCs | 0.1 ms$^{-1}$ | [3] |
| $k_{pre}$ | The efficacy of neurotransmitter release | 3.9 | [3] |
| $b$ | Scaling factor of gamma-distribution | 20 | [3] |
| $k_0$ | The efficacy of neurotransmitter release without astrocytic impact | 3.9 | [3] |
| $b_0$ | Scaling factor of gamma-distribution without astrocytic impact | 20 | [3] |
| $\gamma_1$ | Presynaptic feedback gain describing the influence of astrocytic glutamate on the average amount of released neurotransmitter | 0.1, -0.4 for potentiation and for depression of presynaptic neurotransmitter release, respectively | [3] |
| $\gamma_2$ | Postsynaptic feedforward gain describing the influence of astrocytic D-serine on EPSCs amplitudes | 1 | [3] |
| $\Theta_G$ | Midpoint of neuronal activation | 0.2 | [3] |
| $k_G$ | Slope of neuronal activation | 0.05 | [3] |
| $v_1$ | Max $Ca^{2+}$ channel flux | 6 s$^{-1}$ | [4,5] |
| $v_2$ | $Ca^{2+}$ leak flux constant | 0.11 s$^{-1}$ | [4,5] |
| $v_3$ | Max $Ca^{2+}$ uptake | 2.2 μM s$^{-1}$ | [4,5] |
| $k_3$ | Activation constant for ATP-$Ca^{2+}$ pump | 0.1 μM | [4,5] |
| $a_2$ | $Ca^{2+}$ inhibition constant | 0.2 s$^{-1}$ | [4,5] |
| $d_1$ | Dissociation constant for $IP_3$ | 0.13 μM | [4,5] |
| $d_2$ | Dissociation constant for $Ca^{2+}$ inhibition | 1.049 μM | [4,5] |
| $d_3$ | Receptor dissociation constant for $IP_3$ | 0.9434 μM | [4,5] |
| $d_5$ | $Ca^{2+}$ activation constant | 0.082 μM | [4,5] |
| $v_\beta$ | Maximal rate of $IP_3$ production by | 63 μM s$^{-1}$ | [6] |

| | | | |
|---|---|---|---|
| | PLCβ | | |
| $K_r$ | Glutamate affinity of the receptor | 1.3 μM | [6] |
| $K_p$ | $Ca^{2+}$/PKC-dependent inhibition factor | 10 μM | [6] |
| $K_\pi$ | $Ca^{2+}$ affinity of PKC | 0.6 μM | [6] |
| $v_\delta$ | Maximal rate of $IP_3$ production by PLCδ | 0.02 μM $s^{-1}$ | [6] |
| $K_\delta$ | Inhibition constant of PLCδ activity | 1.5 μM | [6] |
| $K_{PLC\delta}$ | $Ca^{2+}$ affinity of PLCδ | 0.1 μM | [6] |
| $v_{3K}$ | Maximal rate of degradation by $IP_3$-3K | 2 μM $s^{-1}$ | [6] |
| $K_D$ | $Ca^{2+}$ affinity of $IP_3$-3K | 0.7 μM | [6] |
| $K_3$ | IP3 affinity of $IP_3$-3K | 1 μM | [6] |
| $r_{5p}$ | Maximal rate of degradation by IP-5P | 0.04 $s^{-1}$ | [6] |
| $\alpha_1$ | Clearance constant of glutamate released from astrocyte | 10 $ms^{-1}$ | [3] |
| $\alpha_2$ | Clearance constant of D-serine released from astrocyte | 10 $ms^{-1}$ | [3] |
| $[Ca_c]_{th}$ | Threshold intracellular calcium concentration for gliotransmission | 0.19 μM | [9] |
| $k_{1,2}$ | Slope of activation function $H_{1,2}(X)$ | 0.01 | [3] |
| $D_{Ca}$ | Coefficient of $Ca^{2+}$ diffusion | 30 $\mu m^2/s$ | [10] |
| $D_{IP3}$ | Coefficient of $IP_3$ diffusion | 300 $\mu m^2/s$ | [10] |
| $d_{CaER}$ | Coefficient of $Ca^{2+}$ diffusion in ER | 0.001 $s^{-1}$ | [10] |
| $F$ | Faraday constant | 96485 C/mol | |
| $r_{ER}$ | Ratio of the volume of ER to the volume of cytoplasm | 0.15-0.05 see Table 2 | [7,8] |
| $r$ | Radius of the astrocytic compartment | 0.25-8 μm see Table 2 | [7,8] |
| $l$ | Unit length | 1 μm | |

[1] (Izhikevich, 2007)
[2] (Kazantsev, Asatryan 2011)
[3] (Gordleeva et al., 2012)
[4, 5] (De Young & Keizer, 1992; Li & Rinzel, 1994)
[6] (De Pitta M et al., 2009)
[7, 8] (Oschmann et al., 2017, Patrushev et al., 2013).
[9] (Parpura & Haydon, 2000)
[10] (Kang & Othmer, 2009)

**Table 2. Geometry of the astrocytic model**

| **Compartment number (Fig. 2A), N** | **Value** $r_{ER}$ | **Value** $r$, μm |
|---|---|---|
| Soma, 0 | 0.15 | 8 |
| 1, 9, 14, 19, 25, 33, 39 | 0.1 | 4 |

| | | |
|---|---|---|
| 2, 6, 10, 15, 20, 26, 28, 34, 40, 47 | 0.09 | 3 |
| 3, 5, 7, 8, 11, 16, 21, 27, 29, 35, 48, 41 | 0.08 | 2 |
| 4, 12, 17, 22, 30, 36, 42, 49, 50 | 0.07 | 1 |
| 13, 18, 23, 31, 37, 43, 51, 52 | 0.06 | 0.5 |
| 24, 32, 38, 44, 45, 46 | 0.05 | 0.25 |
